# Supplementary material for: Post-COVID-19 syndrome among symptomatic COVID-19 patients: A prospective cohort study in a tertiary care center of Bangladesh
Source: PLoS One. 2021 Apr 8;16(4):e0249644. doi: 10.1371/journal.pone.0249644 (PMC8031743; doi:10.1371/journal.pone.0249644)
Supplement: S1 Protocol — (DOCX) [file pone.0249644.s001.docx]

**Research Protocol**

**Post COVID Syndrome among Symptomatic COVID-19 Patients**

**Principal Investigator**

**Dr. Reaz Mahmud**

**FCPS (Medicine), MD (Neurology)**

**Assistant Professor**

**Department of Neurology**

**Dhaka Medical College, Dhaka**

**
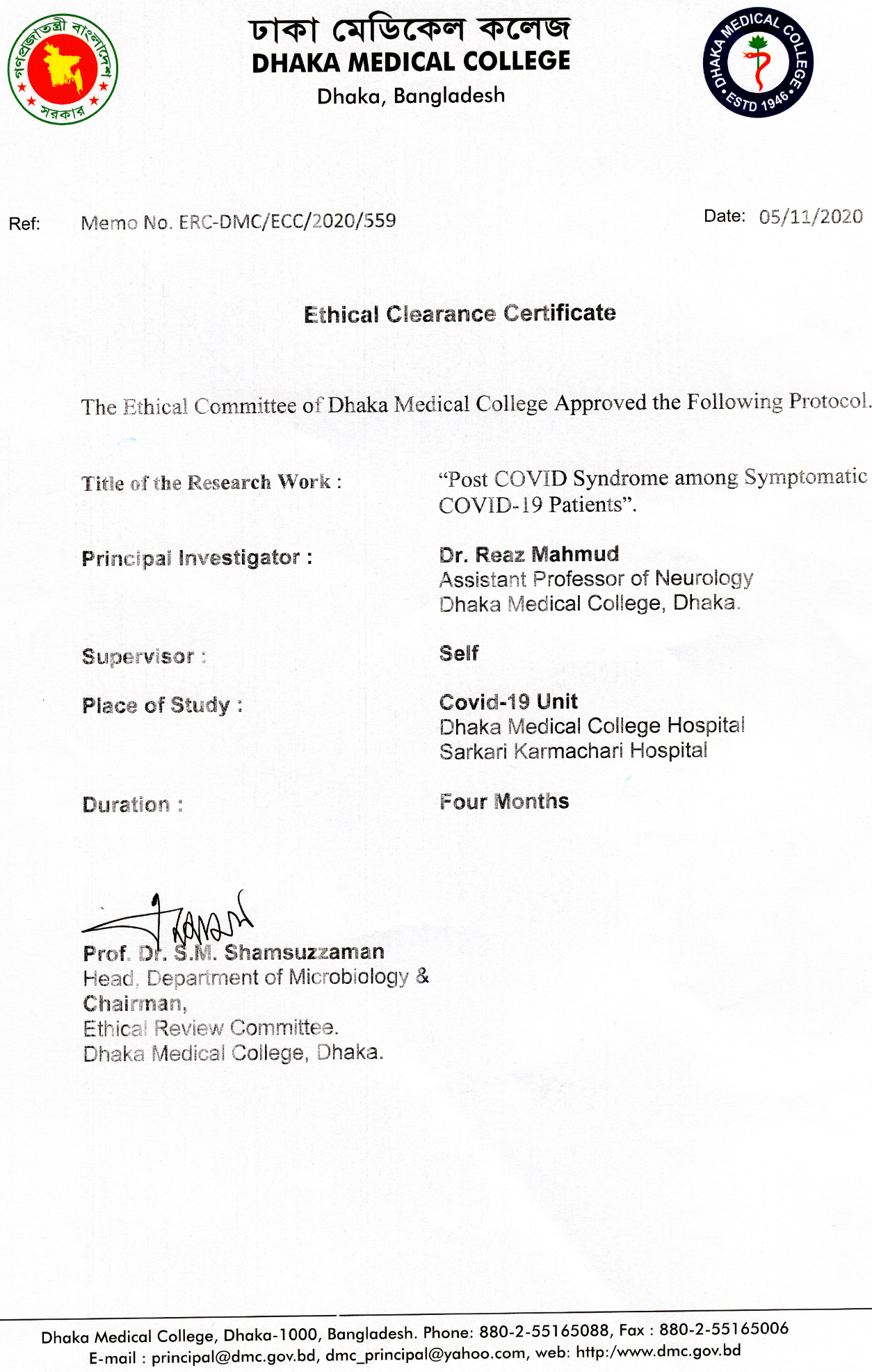
**

**Part A**

**Project Title: Post COVID syndrome among symptomatic COVID-19 patients**

# Principal Investigator:

| Name | Designations & place of posting |
| --- | --- |
| Dr. Reaz Mahmud | Assistant Professor, Department of Neurology, Dhaka Medical College |

2. **Co-Principal investigators:**

| Name | Designations & place of posting |
| --- | --- |
| Professor Mujibur Rahman | Professor and Head, Department of Medicine, Dhaka Medical College |

1. **Co-investigators:**

| Name | Designations& place of posting |
| --- | --- |
| Dr. S. K Jakaria Been Sayeed | Indoor Medical Officer, Department of Medicine, Dhaka Medical College. |
| Dr.MD. Shahidul Islam | Junior Consultant, Medecine, Sarkari Karmachari Hospital, Dhaka. |
| Dr. Mohammad Aftab Rassel | Medical officer, OSD, MD Thesis part student, Department of Neurology, Dhaka Medical College |
| Dr. Farhana Binte Monayem | Medical Officer, Sarkari Karmachari Hospital,Dhaka. |
| DR. Mohammed Monirul Islam | Assistant Surgeon, MoHFW, Dhaka, Bangladesh |

1. Place of the study/Institution(s):

COVID-19 Unit, Dhaka Medical College Hospital and Sarkari Karmachari Hospital.

1. Sponsoring/Collaborating Agencies:

None

1. Duration:

04 months

1. Date of Commencement:

July 2020

1. Date of Completion:

August 2020

1. Total Cost: Not estimated

10. Other Support for Proposed Research:

(1) Is this research project being No

supported by any other source?

(2) Has an application for funding of No

this project been submitted to any

other organization(s)?

11. Date of Submission : 04-10-2020

12. Signature of Principal Investigator(s) :

| Name | Designations & place of posting | Signature |
| --- | --- | --- |
| Dr. Reaz Mahmud | Assistant Professor, Department of Neurology, Dhaka Medical college |  |

**PART – B**

**PRINCIPAL INVESTIGATOR(S) INFORMATION SHEET**

1. (i) Name :**Dr. Reaz Mahmud**

(ii) Designation: Assistant Professor, Neurology

(iii) Official Address with telephone: Dhaka Medical College Hospital, Dhaka.

Phone: 01912270803

(iv) Present Residential Address with telephone: Road # 07, House # 13

Abdullah bagh, Uttar Badda, Badda Dhaka.

1. **Academic Background:**

| Degree | University | Field | Year |
| --- | --- | --- | --- |
| MBBS | Dhaka University |  | May,2003 |
| FCPS | BCPS | Medicine | January,2013 |
| MD | Dhaka University | Neurology | June,2015 |

1. **Field of Specialty:**

Medicine, Neurology, Research Methodology and Data Analysis

1. **Membership:** Bangladesh Society of Medicine, Society of Neurologists Bangladesh, International Headache society, Asia Pacific Headache Society.
2. **(a) Research Experience :**

| 1. **Association between Hypertensive retinopathy and Lacunar stroke.** | Done for FCPS Dissertation |
| --- | --- |
| 2. **Risk Factors and Morphological Differences of Ruptured Saccular Aneurysm in different sites of Anterior Circulation in Patients presenting with Subarachnoid Haemorrhage** | Done for MD thesis |
| 3**. Recent sensitivity Pattern of Salmonella Typhi in a private hospital.** | Done for Research |
| 4. A Randomized, Double-Blind Placebo Controlled Clinical Trial of Ivermectin plus Doxycycline for the Treatment of Confirmed Covid -19 Infection | Completed Research |

**(b) Other Experience:**

**Teaching:**

1. Assistant Registrar cardiology NICVD, 03-08-09 to 21-06-10
2. Assistant Registrar Medicine, Faridpur Medical college Hospital, Faridpur 23-06-10 to 31-06-12

**Administration:**

Work as a Departmental Head, Critical Care Medicine, Sarkari Karmachari Hospital from 01-11-2016 to 04-12-2019

5. **Number of Scientific Publications:**

| Sl No | National/  International | Original/  review | Authorship | Reference |
| --- | --- | --- | --- | --- |
| 1 | National | Original | Co-author | Ali M Y, Mahmud R. A case report on Lepra Reaction Type II. Faridpur Medical college journal 2012; 2 : 93-97 |
| 2 | National | Original | Author | Mahmud R, Ali MY, Islam MS, Shanewaz S, Rabbani G, Manayem FB. Association Between Hypertensive Retinopathy and Lacunar Infarct- A Study in Faridpur Medical College Hospital. DCIMC J 2016; 3(2):27-33 |
| 3 | National | Original | Co-author | Saha R, Mahmud R, Hossain MZ,Sarker PK. Families with Neurocutaneous Syndrome:Report of two cases.Dhaka Med Coll J 2013; 22(1): 102-107. |
| 4 | National | Original | Author | Mahmud R, Habib M, Uddin S,Risk Factors and Morphological Differences of Ruptured Saccular Aneurysm in Different Sites of Anterior Circulation in Patients Presenting with Subarachnoid Haemorrhage. Journal of National Institute of Neurosciences Bangladesh. January 2017; 3(1):21-28. |
| 5 | National | Original | Author | Islam K, Mahmud R. Recent sensitivity Pattern of Salmonell Typhi in a private hospital.J Medicine 2018; 19: 15-17 |
| 6 | National | Original | Author | Mahmud R, Habib M. Huntington's Disease with Retinitis Pigmentosa- a Case Report. Faridpur Med. Coll. J 2017; 12(1):50-52. |
| 7 | National | Original | Co-Author | Ghose, S., Ahmed, K. G., Chowdhury, A., Hasan, A., Saha, K., Mahmud, R., Joy, N., Biswas, R., Sarkar, M. S., Rahman, M. M., Sina, H., Arifuzzaman, M., Alam, I., Hossain, M. M., Karim, A., & Habib, M. (2018). Assessment of Initial Stroke Severity by National Institute Health Stroke Scale (NIHSS) Score at Admission. *Journal of Dhaka Medical College*, *26*(2), 90-93. https://doi.org/10.3329/jdmc.v26i2.38765 |

Signature of Principal Investigator

**PART - C**

**Project Title: Post COVID syndrome among symptomatic COVID-19 patients**

**Summary**:

Since December 2019 COVID-19 is dominating the life of the people of this universe. Its behavior also varied among the nations of the universe. The patients presented with varied symptoms, severity and outcome. Most of the patients recovered within 14 days. But several studies revealed persistent of the symptoms among the COVID affected patients months after recovery. The definitions and symptoms of these post COVID, long COVID or Persistent COVID is yet to be determined. There is no consensus regarding this aspect of COVID.

So to observe the various symptoms and their spectrum we would like to conduct this prospective study. We defined all these post COVID symptoms under a common umbrella post COVID syndrome. This will to label the condition and future treatment planning.

The primary outcome would be the varied symptoms developed after apparent clinical improvement.

The study will be a hospital based Prospective Cohort study. It will be conducted in the Covid-19 unit Dhaka Medical college Hospital from 01 June 2020 to 10 August 2020. Institutional ethical committee approval was obtained. Patients presented in the triage and inpatient department of Dhaka Medical College, will be enrolled according to defined inclusion and exclusion criteria. Informed consent was taken from every patients. The Recruited patients were followed up for at least 1 month after clinical recovery and /or virologic clearance. . The data was analyzed by using SPSS 20 software. To observe the relationship binary logistic regression, Relative risk ratio, 95%Confidence interval were used.Result of the study and statistical analysis will be presented by tables, figures, graphs, diagrams, charts and photographs. All these will have own legends (i.e. title) and will be serially numbered. Discussion will be done on the basis of result obtained from the study and comparing with similar studies done at home and abroad. Summarization will be drawn after discussion. Conclusion will be drawn depending upon the results and discussion.

**PART - D**

**Introduction:**

Since the first recognition of COVID-19 on 31 December, the virus is dominating the life of every people of this universe. The clinical presentation of COVID-19 ranges from asymptomatic to fulminant and fatal cases. Severe cases of infection can develop pneumonia, acute respiratory distress syndrome, sepsis and/or multiple organ failure which are not unique to coronavirus^1^.

Death is due to pneumonia and possibly hyper-inflammation associated with cytokine storm syndrome^2^. Complications can include: Pneumonia, Organ failure in several organs, Myocarditis, acute MI, ARDS, Blood clots, acute kidney injury and Additional viral and bacterial infections^3^.

Most of the patient recovered within 14 days. But this is not the end of the sufferings. 87.4% patients reported persistence of at least 1 symptom^4^. In UK a smartphone app based study revealed that in about 10% of the patient symptoms persisted after 3 weeks, and some may have symptoms for months^5^.In USA it was nearly 90% of hospitalized patients who recovered from COVID-19 reported persistence of at least one symptom 2 months after discharge^6^.

There is no consensus about the definition of post Covid syndrome. For the purpose of this article we define the post Covid syndrome as

1. Persistence of illness signs and symptoms (except fever, respiratory distress, hypoxia) beyond the virologic clearance.
2. New development of a symptoms within 1 month after initial clinical and virologic cure, the etiology of which postulated to be viral infection.
3. Exaggeration of previously experienced chronic disease (i.e. Migraine, Mental disorder, bronchial asthma, Rheumatologic disorders) after the recovery from Covid 19.

The mechanism is not established yet. Previous experience about SARS, which was also a corona virus revealed that post viral fatigue syndrome/Myalgic Encephalomyelitis was the most common symptoms developed in that disease. It was found that the virus reach to hypothalamus via olfactory pathway and disturb its lymphatic drainage. It leads to build up of pro-inflammatory cytokine, interleukins, and interferon gamma^8, 9^ within the substance of hypothalamus which leads to development of the feature of post viral fatigability.

This study aim to find out the Incidence, types and association of different variables in development of the post Covid syndrome among the Covid affected patients.

**2. OBJECTIVES:**

**General objectives:**

To observe the types of the post Covid symptoms among the confirmed COVID-19 cases.

**Specific objectives:**

1. To observe the incidence of the post Covid syndrome
2. To observe the association of different variables in development of the post Covid syndrome

**3. RATIONALE:**

Covid-19 is an emergent pandemic, threatens the life of millions of the people throughout the globe. The severity, outcome and susceptibility varies among the nations, race and individuals. Significant number of the patients also suffering from the post COVID sequel. For their proper management identification and proper treatment is a must. This is a prospective cohort study. It will help to detect the incidence and the risk factors for developing the post COVID Syndrome.

1. **METHODOLOGY:**

Study type: Prospective cohort

Sample size:

Formula n=z^2^pq/d^2,^

Here, z=1.96 (at 95% confidence level)^,^ p=50%, as prevalence of post COVID syndrome is not known in Bangladesh^,^ q= (100-p) =50. Here d is absolute error, 5% was considered.

So, sample size, n= (1.96)^2^X50X50/5^2^=384.

For the convenience 400 will be taken.

**Sampling: Consecutive sampling.**

Estimated study date: June, 2020

Estimated primary completion date, August 2020

Estimated study completion date: September, 2020

**Inclusion Criteria:**

- At least 18 years of age
- COVID-19 infection, confirmed by polymerase chain reaction (PCR) test
- Mild to severe COVID-19 infection
- Able to provide informed consent

**Exclusion Criteria:**

- Not willing to participate.
- Critical COVID-19 patients admitted in ICU

**Research instruments:**

1. Informed consent form.

2. Case record form

**Primary Outcome Measure:**

- 1. Development of the post COVID symptoms

[Time Frame: 1 months]

**Secondary Outcome Measure:**

1. Types of the post COVID symptoms

Time Frame: 1 month]

**Data collection technique:**

Data will be collected by assigned trained data collectors (Physician).

Patient will be enrolled according to defined inclusion and exclusion criteria in the current research. Informed written consent will be obtained from the participants. Each participants participating in the trial will be uniquely identified, and information such as his name, address is recorded in the trial 'subject number list'.

**Data analysis**:

The data was analyzed by using SPSS 20 software. To observe the relationship binary logistic regression, Relative risk ratio, 95%Confidence interval were used.

**Observation and Results:**

Result of the study and statistical analysis will be presented by tables, figures, graphs, diagrams, charts and photographs. All these would have own legends (i.e. title) and will be serially numbered.

**Discussion:**

Discussion will be done on the basis of result obtained from the study and comparing with similar studies done at home and abroad.

**Summary:**

Summarization will be drawn after discussion.

**Conclusion:**

Conclusion would be drawn depending upon the results and discussion

**Conflict of interest:** None

**Operational definition:**

**(According to WHO and National guideline)**

**Confirmed Covid-19:**

Cases with positive RT-PCR for Covid 19 irrespective of symptoms**.**

**Uncomplicated (mild) Illness**

These patients usually present with symptoms of an upper respiratory tract viral infection, including mild fever, cough (dry), sore throat, nasal congestion, malaise, headache, muscle pain, or malaise. Signs and symptoms of a more serious disease, such as dyspnea, are not present.

**Moderate Pneumonia**

Respiratory symptoms such as cough and shortness of breath (or tachypnea in children) are present without signs of severe pneumonia.

**Severe Pneumonia**

Fever is associated with severe dyspnea, respiratory distress, tachypnea (> 30 breaths/min), and hypoxia (SpO2 < 90% on room air). However, the fever symptom must be interpreted carefully as even in severe forms of the disease, it can be moderate or even absent. Cyanosis can occur in children. In this definition, the diagnosis is clinical, and radiologic imaging is used for excluding complications.

**Acute Respiratory Distress Syndrome (ARDS)**

The diagnosis requires clinical and ventilatory criteria. This syndrome is suggestive of a serious new-onset respiratory failure or for worsening of an already identified respiratory picture. Different forms of ARDS are distinguished based on the degree of hypoxia. The reference parameter is the PaO2/FiO2:

- Mild ARDS: 200 mmHg < PaO2/FiO2 ≤ 300 mmHg. In not-ventilated patients or in those managed through non-invasive ventilation (NIV) by using positive end-expiratorypressure (PEEP) or a continuous positive airway pressure (CPAP) ≥ 5 cmH2O.
- Moderate ARDS: 100 mmHg < PaO2/FiO2 ≤ 200 mmHg.
- Severe ARDS: PaO2/FiO2 ≤ 100 mmHg.

When PaO2 is not available, a ratio SpO2/FiO2 ≤ 315 is suggestive of ARDS.

Chest imaging utilized includes chest radiograph, CT scan, or lung ultrasound demonstrating bilateral opacities (lung infiltrates > 50%), not fully explained by effusions, lobar, or lung collapse.

**Clinical improvement Criteria**

1. Body temperature remains normal for at least 3 days (ear temperature is lower than 37.5 °C).

2. Respiratory symptoms are significantly improved.

3. Lung imaging shows obvious improvement in lesions.

4. There is no co-morbidities or complications which require hospitalization.

5. SpO2, >93% without assisted oxygen inhalation.

**5. UTILIZATION OF RESULTS:**

This study will observe the benefit of combined Doxycycline and Ivermectin on confirmed covid 19 patient. The study result will be compared with other trials in home and abroad.

**8. FLOW CHART of Study workup:**

| Sl No | **Activities** |  |  |  |  |  |  |
| --- | --- | --- | --- | --- | --- | --- | --- |
|  |  | **1^st^** | **2^nd^** | **3^rd^** | **4^th^** | **5^th^, 6 ^th^, 7 ^th^** | **8^th^** |
| 1 | **Recruitment and training of the field staff** |  |  |  |  |  |  |
| 2 | **Pretesting and finalization of the questionnaire** |  |  |  |  |  |  |
| 3 | **Consultative meeting** |  |  |  |  |  |  |
| 4 | **Data collection** |  |  |  |  |  |  |
| 5 | **Data entry and editing** |  |  |  |  |  |  |
| 6 | **Data analysis and draft report writing** |  |  |  |  |  |  |
| 7 | **Dissemination of Results** |  |  |  |  |  |  |

1. **ETHICAL IMPLICATIONS**

The following points will be considered during the study:

1. Patients (subjects) and key relatives were clearly informed about the scope and limitation of the study.

2. Written consent will be obtained from the patients (subjects) or from parents if patient (subject) is unable to give reliable information.

3. Confidentiality of the patients (subjects) about personal information was strictly maintained.

4. The study will not be causing any environmental hazard.

**REFERENCES:**

1. Rodriguez-Morales AJ, Cardona-Ospina JA, Gutie´rrez-Ocampo E, Villamizar-Pen˜a R, Holguin-Rivera Y, Escalera-Antezana JP, et al. Clinical, laboratory and imaging features of COVID-19: a systematic review and meta-analysis. Travel Med Infect Dis. 2020; 34:101623.
2. Mehta P, McAuley DF, Brown M, Sanchez E, Tattersall RS, Manson JJ. COVID-19: consider cytokine storm syndromes and immunosuppression. Lancet 2020;395 (10229):1033–4.
3. Zheng, KI, Feng, G, Liu, W‐Y, Targher, G, Byrne, CD, Zheng, M‐H. Extrapulmonary complications of COVID‐19: A multisystem disease? *J Med Virol*. 2020; 1– 13. <https://doi.org/10.1002/jmv.26294>.
4. Carfì A, Bernabei R, Landi F, for the Gemelli Against COVID-19 Post-Acute Care Study Group. Persistent Symptoms in Patients after Acute COVID-19. JAMA. Published online July 09, 2020. doi:10.1001/jama.2020.12603
5. COVID Symptom Study. How long does COVID-19 last? 2020 [internet publication}
6. Carfì A, Bernabei R, Landi F, et al. Persistent symptoms in patients after acute COVID-19. JAMA. 2020 Jul 9 [Epub ahead of print].
7. Tenforde MW, Kim SS, Lindsell CJ. Symptom duration and risk factors for delayed return to usual health among outpatients with COVID-19 in a multistate health care systems network: United States, March-June 2020. MMWR Morb Mortal Wkly Rep. 2020 Jul 31;69(30):993-8
8. Moldofsky H, Patcai J. Chronic widespread musculoskeletal pain, fatigue, depressionand disordered sleep in chronic post-SARS syndrome; a case-controlled study. BMC Neurol 2011;11:1–7.
9. Holmes TH, Anderson JN, et al. Cytokine signature associated with disease severity in World Health Organization. GCM teleconference – Note for the Records. 10 January 2020. Subject: Pneumonia in Wuhan, China. Available from: https: // www. who. int/ blueprint / 10-01-2020-nfr-gcm.pdf?ua=.

**Abstract summary for Ethical Committee**

This hospital based Experimental study will be carried out in COVID-19 Unit, Dhaka Medical College Hospital and Sarkari Karmachari Hospital for the duration of 4 months. Consecutive confirmed COVID-19 patients with mild to moderate severity whom will be enrolled from June, 2000 to august, 2000. Total 400 cases will be enrolled for the research.

1. By the following under mentioned steps confidentially will be maintained:

- Research data will be coded
- Data will be stored in a locked cabinets
- Only research personnel will be allowed to access data
- There is no physical, psychological, social and legal risk during physical examination. Proper consent will be taken.
- For safeguarding confidentially and protecting anonymity each of the patient will be given and ID no.
- A signed informed consent will be taken from the patient/patients guardians convincing that privacy of the patient will be maintained and he/she will not be compensated for loss of work time if they want.
- A data should (enclosed) be prepared for which a short interview of 15-30 minutes will be required
- Use of hospital records (indoor) will be needed to fill up the patients’ data sheet.

2. Consent form will be a written statement

1. A brief interview regarding study variables will be collected from the participants.

**Circle the appropriate answer to each of the following**

**(If not Applicable write NA).**

| **1. Source of population:** |  |  | **4. Are subjects clearly informed about** |  |  |
| --- | --- | --- | --- | --- | --- |
| a) Patients | Yes | No | a) Nature and purposes of study | Yes | No |
| b) Healthy subjects | Yes | No | b) Procedures to be followed including alternatives used | Yes | No |
| c) Minors or persons under guardianship | Yes | No | c) Physical risks | Yes | No |
| **2. Does the study involve:** |  |  | d) Private questions | Yes | No |
|  |  |  | e) Invasion of the body | NA | |
| a) Physical risks to the subject | Yes | No | f) Benefits to be derived | Yes | No |
| b) Social Risks | Yes | No | g) Right to refuse, to participate or to withdraw from study | Yes | No |
| c) Psychological risks to subjects | Yes | No | h) Confidential handing of data | Yes | No |
| d) Discomfort to subjects | Yes | No | i) Compensation where there are risks or loss of working time or privacy is involved in any particular procedure | NA | |
| e) Invasion of the Body | Yes | No |  |  |  |
| f) Invasion of privacy | Yes | No |  |  |  |
| g) Disclosure of information damaging to subjects or others | Yes | No |  |  |  |
| **3. Does the study involve:** |  |  | **5. Will signed consent form/verbal Consent be required:** |  |  |
| a) Use of records, (hospital, medical, death, birth or other). | Yes | No | a) From Subject | Yes | No |
| b) Use of fetal tissue or aborts | Yes | No | b) From parent or guardian (if Subjects are minors) | NA | |
| c) Use of organs or body fluids | Yes | No | **6. Will precautions be taken to protect anonymity of subjects.** | Yes | No |

**Patient Information Sheet**

(For the patients/respondents)

*(Please read the handout in front of patient/respondent and explain in local language and understandable way).*

The objective of this handout is to give you necessary information that will help you to take decision whether you will participate in this research work or not.

1. About the study:

To assess the incidence, type and association of Post COVID syndrome among the confirmrd COVID-19 cases. This research work will be conducted by the Department of Medicine, Dhaka Medical College, Dhaka. We want to include you as a study participant after receiving a written consent from you. I will explain you in a moment what are the components and your role in the study.

2. Purpose of the study:

Find out the incidence, type and association of Post COVID syndrome among the confirmrd COVID-19 cases

3. Confidentiality:

The information that we will collect from this research project will be kept confidential unless permitted by you. Information that will be collected from this study will only be used for research purpose. Your personal information will not be disclosed to anyone other than the investigators.

4. Right to refuse or withdraw:

You have all the right to refuse to participate in this study if you do not wish to do so. Refusing to participate will not affect your treatment in any way. You may stop participating in this study at any time you wish.

5. Incentives:

You will not be provided any incentives to take part in this research. You will be given honorium and conveyance expenditure if you are to come for this research work.

6. Risks and discomforts:

There is a slight risk that you may share some personal and confidential information by chance or that you may feel uncomfortable about some of the topics. However, we do not wish this to happen. You may refuse to give answer to any question or any portion of it if you need to do so.

7. Benefits:

You might not get direct benefit from this study. You will get appropriate treatment in this hospital after the diagnosis of your disease. Your participation is likely to help us to acquire more knowledge about this disease which may be of benefit to other patients of our country.

8. Procedure of research:

If you agree, we will enroll you as a study participant and will adopt the following procedures for your participation-

i. We will take signature/thumb impression in the attached consent form in

duplicate and a copy will be returned to you.

ii. We will ask you some questions to fill in a printed Case Record Form.

iii. You will be examined physically for the sake of this study.

If you agree to participate in this study, please sign the attached consent form.

**INFORMED CONSENT FORM**

I, Mr/Mrs/Miss ……………………………………………., hereby giving informed consent willingly to participate in the study to be done by Dr. Reaz Mahmud. I agree to participate in the study voluntarily without any prejudice. I am fully convinced that during study I will not suffer from any serious physical or psychological problems. I am also informed that this study was carried out in the developed countries safely and my participation will bring fruitful result that will be beneficial for most patients in our country. I have right to withdraw myself from this study at any time. I shall not receive any financial benefit. I have understood that my personal information, medical records & laboratory tests will be kept strictly confidential & will be used for research purpose only.

Signature/Thumb impression of participant/Guardian:………………………….

Date: ………………………………

Name: …………………………………………………

Address: ………………………………………………

………………………………………………………….

………………………………………………………….

Signature of witness Signature of Researcher

Date: Date:

Name of witness:

**PART-H**

**Informed Consent Form**

Project Title: **Post COVID syndrome among symptomatic COVID-19 patients**

# AeMwZµ‡g m¤§wZcÎ–

**GB m¤§wZc‡Îi D‡Ïk¨ nj Avcbv‡K cÖ‡qvRbxq Z_¨ cÖ`vb Kiv, †h Z_¨¸‡jv Avcbv‡K wm×všÍ wb‡Z mvnvh¨ Ki‡e, Avcwb GB M‡elYvq AskMÖnb Ki‡eb wKbv ?**

**D‡Ïk¨ c×wZ:**

**GB M‡elYvwU XvKv‡gwW‡Kj K‡jR nvmcvZvj ‡KvwfW-19 BDwbU Gi ZË¡veav‡b cwiPvjZ n‡e| †KvweW-19 G AvµvšÍ †ivMxM‡bi Dci cwiPvwjZ n‡e| cÖv_wgK fv‡e AskMÖnbKvix Mb‡K cÖkœ Kiv n‡e|**| **†KvweW-19 G AvµvšÍ †ivMxMb my¯’ nevi ci wK wK Dcm‡©M AvµvšÍ nb M‡elKMi vbY„q K**i‡eb| M‡elYvjä Z_¨ †`‡ki wecyj msL¨K‡KvwfW-19 cieZ©x DcmM© &I Zvi **SzwK vbY„‡q** Kv‡R jvM‡e|

**M‡elYvi SuywK:**

GB M‡elYvq AskMÖnYKvix‡`i ‡Kvb **SzwK bvB|**

**M‡elbvq AskMÖn‡Yi myweavw`:**

GB M‡elYvq AskMÖnY Ki‡j Avcwb e¨w³MZfv‡e jvfevb n‡Z cv‡ib| Gi gva¨‡g fwel¨‡Z **†KvweW-19 G AvµvšÍ nevi SzwK m¤ú‡K©** civgk© †`qv m¤¢e n‡e| GB M‡elbv evsjv‡`‡ki wPwKrmK‡`i **†KvweW-19** m¤ú‡K© Av‡iv Rvb‡Z mnvqZv Ki‡e|

**weKí:**

G M‡elYvq AskMÖnY Kiv wKsev bv Kivi e¨vcv‡i ev AskMÖnY Kivi ci †h †Kvb mgq Avcwb wb‡R‡K M‡elYv †_‡K mwi‡q wb‡Z cvi‡eb|

**LiP:**

GB M‡elYvq AskMÖn‡Yi Rb¨ wba©wiZ wPwKrmv e¨q e¨wZZ Avcbvi AwZwi³ LiP bvB ev Avcbv‡K †Kvb UvKv cqmv †`qv n‡e bv|

**‡MvcbxqZv:**

M‡elYvPjvKvjxb I cieZx©‡Z mKj Z_¨ K‡Vvifv‡e †Mvcb ivLv n‡e| cieZx©‡Z d‡jvAvc I Abymib cÖwµqvi Rb¨ Avcbv‡K GKwU AvBwW b¤^i †`Iqv n‡e| Avcbvi AvBwW b¤^i m¤^wjZ me ai‡bi KvMRc‡Î Avcbvi bvg I wVKvbv ewm‡q dvBwjs †Kwe‡b‡U Zvjve× _vK‡e|

**‡¯^”Qvg~jK AskMÖnY:**

GB M‡elYvq Avcbvi AskMÖnY m¤ú~Y© †¯^”Qvg~jK| Avcwb M‡elYvq AskMÖn‡Y A¯^xK…wZ Rvbv‡Z cv‡ib A_ev M‡elYvPjvKvjxb †h †Kvb mg‡q M‡elYv †_‡K wb‡R‡K cÖZ¨vnvi K‡i wb‡Z cv‡ib| Zv‡Z Avcbvi wPwKrmvi †Kvb ZviZg¨ n‡e bv| GB d‡g© ¯^v¶i Ki‡j Avcbvi AvBbMZ †Kvb AwaKvi Le© n‡e bv|

**cÖkœvejx:**

hw` Avcbvi †Kvb cÖkœ _v‡K Z‡e `qv K‡i wRÁvmv Ki‡eb| Avgiv Gi DËi cÖ`vb Kivi h_vh_ †Póv Kie| hw` fwel¨‡Z Avcbvi AwZwi³ †Kvb cÖkœ _v‡K Zvn‡j M‡elYviZ wPwKrm‡Ki mv‡_ †hvMv‡hvM Ki‡Z cv‡ib|

**Dr. Reaz Mahmud Mobile: 01912270803**

**m¤§wZi ¯^xKv‡ivw³:**

Avwg M‡elbvq wb‡qvwRZ wPwKrmK-Gi mv‡_ (whwb Avgvi kvixwiK cix¶v Ki‡eb) GB M‡elYv wb‡q Av‡jvPbvq mš‘wó cÖKvk KiwQ| Avwg GUv ey‡SwQ †h M‡elYvq AskMÖnb †¯^”Qvg~jK Ges Avwg †h †Kvb mgq †Kvb eva¨evaKZv QvovB M‡elYv †_‡K wb‡R‡K weiZ ivL‡Z cvwi| Avwg Dc‡iv³ kZ©¸‡jv c‡owQ/Avgvi m¤§y‡L cwVZ n‡q‡Q Ges †¯^”Qvq M‡elYvq AskMÖnY Ki‡Z m¤§wZ Ávcb KiwQ|

| ¯^v¶vrKvixi MÖnYKvixi ¯^v¶i:  ZvwiL: | Ask MÖnYKvixi ¯^v¶i/  e„×v½ywji Qvc | ¯^v¶xi ¯^v¶i |
| --- | --- | --- |

Project Title: **Post COVID syndrome among symptomatic COVID-19 patients**

**Case record form**

**Patient ID**: ……………

**Demography**

1. Name: 2. Age: 3. Sex: M / F

4. Address:

5. **Epidemiological link**: Y / N

5. Mobile No:

6. Residency: Urban / Rural

7. Smoker: Y / N

8. Marital Status: Married / Single

9. Education: Literate / Illiterate

Date of starting symptoms

Date of becoming Covid positive

Hospital Admission Date

**Clinical Feature**

| Trait |  | Follow-up  Yes-1, No-2 | | | | | | | | | | | | |
| --- | --- | --- | --- | --- | --- | --- | --- | --- | --- | --- | --- | --- | --- | --- |
|  | On Admission | D2 | D3 | D4 | D-5 | D-6 | D-7 | D-8 | D-9 | D-10 | D-11 | D-12 | D-13 | D-14 |
| Fever | Yes No |  |  |  |  |  |  |  |  |  |  |  |  |  |
| Cough | Yes No |  |  |  |  |  |  |  |  |  |  |  |  |  |
| Running nose | Yes No |  |  |  |  |  |  |  |  |  |  |  |  |  |
| Sputum | Yes No |  |  |  |  |  |  |  |  |  |  |  |  |  |
| Respiratory distress | Yes No |  |  |  |  |  |  |  |  |  |  |  |  |  |
| Sore throat | Yes No |  |  |  |  |  |  |  |  |  |  |  |  |  |
| Hoarseness of voice | Yes No |  |  |  |  |  |  |  |  |  |  |  |  |  |
| Chest pain | Yes No |  |  |  |  |  |  |  |  |  |  |  |  |  |
| Diarrhoea | Yes No |  |  |  |  |  |  |  |  |  |  |  |  |  |
| Vomiting | Yes No |  |  |  |  |  |  |  |  |  |  |  |  |  |
| Anosmia | Yes No |  |  |  |  |  |  |  |  |  |  |  |  |  |
| Anorexia | Yes No |  |  |  |  |  |  |  |  |  |  |  |  |  |
| Headache | Yes No |  |  |  |  |  |  |  |  |  |  |  |  |  |
| Confusion | Yes No |  |  |  |  |  |  |  |  |  |  |  |  |  |

**Subsequent follow up**

| Trait | Follow-up  Yes-1, No-2 | | | | | | | | | | | | | | | |
| --- | --- | --- | --- | --- | --- | --- | --- | --- | --- | --- | --- | --- | --- | --- | --- | --- |
|  | Day-15 | D16 | D17 | D18 | D-19 | D-20 | D-21 | D-21 | D-23 | D-24 | D-25 | D-26 | D-27 | D-28 | D-29 | D-30 |
| Fever |  |  |  |  |  |  |  |  |  |  |  |  |  |  |  |  |
| Cough |  |  |  |  |  |  |  |  |  |  |  |  |  |  |  |  |
| Running nose |  |  |  |  |  |  |  |  |  |  |  |  |  |  |  |  |
| Sputum |  |  |  |  |  |  |  |  |  |  |  |  |  |  |  |  |
| Respiratory distress |  |  |  |  |  |  |  |  |  |  |  |  |  |  |  |  |
| Sore throat |  |  |  |  |  |  |  |  |  |  |  |  |  |  |  |  |
| Hoarseness of voice |  |  |  |  |  |  |  |  |  |  |  |  |  |  |  |  |
| Chest pain |  |  |  |  |  |  |  |  |  |  |  |  |  |  |  |  |
| Diarrhoea |  |  |  |  |  |  |  |  |  |  |  |  |  |  |  |  |
| Vomiting |  |  |  |  |  |  |  |  |  |  |  |  |  |  |  |  |
| Anosmia |  |  |  |  |  |  |  |  |  |  |  |  |  |  |  |  |
| Anorexia |  |  |  |  |  |  |  |  |  |  |  |  |  |  |  |  |
| Headache |  |  |  |  |  |  |  |  |  |  |  |  |  |  |  |  |
| Confusion |  |  |  |  |  |  |  |  |  |  |  |  |  |  |  |  |

| Trait | Follow-up  Yes-1, No-2 | | | | | | | | | | | | | | | |
| --- | --- | --- | --- | --- | --- | --- | --- | --- | --- | --- | --- | --- | --- | --- | --- | --- |
|  | Day-31 | D32 | D33 | D34 | D-35 | D-36 | D-37 | D-38 | D-39 | D-40 | D-41 | D-42 | D-43 | D-44 | D-45 | D-46 |
| Fever |  |  |  |  |  |  |  |  |  |  |  |  |  |  |  |  |
| Cough |  |  |  |  |  |  |  |  |  |  |  |  |  |  |  |  |
| Running nose |  |  |  |  |  |  |  |  |  |  |  |  |  |  |  |  |
| Sputum |  |  |  |  |  |  |  |  |  |  |  |  |  |  |  |  |
| Respiratory distress |  |  |  |  |  |  |  |  |  |  |  |  |  |  |  |  |
| Sore throat |  |  |  |  |  |  |  |  |  |  |  |  |  |  |  |  |
| Hoarseness of voice |  |  |  |  |  |  |  |  |  |  |  |  |  |  |  |  |
| Chest pain |  |  |  |  |  |  |  |  |  |  |  |  |  |  |  |  |
| Diarrhoea |  |  |  |  |  |  |  |  |  |  |  |  |  |  |  |  |
| Vomiting |  |  |  |  |  |  |  |  |  |  |  |  |  |  |  |  |
| Anosmia |  |  |  |  |  |  |  |  |  |  |  |  |  |  |  |  |
| Anorexia |  |  |  |  |  |  |  |  |  |  |  |  |  |  |  |  |
| Headache |  |  |  |  |  |  |  |  |  |  |  |  |  |  |  |  |
| Confusion |  |  |  |  |  |  |  |  |  |  |  |  |  |  |  |  |

**Obsrvation**

| Trait |  | Follow-up  Yes-1, No-2 | | | | | | | | | | | | |
| --- | --- | --- | --- | --- | --- | --- | --- | --- | --- | --- | --- | --- | --- | --- |
|  | On Admission | D2 | D3 | D4 | D-5 | D-6 | D-7 | D-8 | D-9 | D-10 | D-11 | D-12 | D-13 | D-14 |
| Temperature |  |  |  |  |  |  |  |  |  |  |  |  |  |  |
| Pulse rate |  |  |  |  |  |  |  |  |  |  |  |  |  |  |
| BP |  |  |  |  |  |  |  |  |  |  |  |  |  |  |
| Respiratory Rate |  |  |  |  |  |  |  |  |  |  |  |  |  |  |
| Oxygen saturation |  |  |  |  |  |  |  |  |  |  |  |  |  |  |
| Co- Morbidity | DM/HTN/ IHD/ HF/ CKD/CLD/Asthma/COPD/Malignancy/ CTD | | | | | | | | | | | | | |

**Subsequent observation:**

| Trait | Follow-up  Yes-1, No-2 | | | | | | | | | | | | | | | |
| --- | --- | --- | --- | --- | --- | --- | --- | --- | --- | --- | --- | --- | --- | --- | --- | --- |
|  | Day 15 | D16 | D17 | D18 | D-19 | D-20 | D-21 | D-22 | D-23 | D-24 | D-25 | D-26 | D-27 | D-28 | D-29 | D-30 |
| Temperature |  |  |  |  |  |  |  |  |  |  |  |  |  |  |  |  |
| Pulse rate |  |  |  |  |  |  |  |  |  |  |  |  |  |  |  |  |
| BP |  |  |  |  |  |  |  |  |  |  |  |  |  |  |  |  |
| Respiratory Rate |  |  |  |  |  |  |  |  |  |  |  |  |  |  |  |  |
| Oxygen saturation |  |  |  |  |  |  |  |  |  |  |  |  |  |  |  |  |

| Trait | Follow-up  Yes-1, No-2 | | | | | | | | | | | | | | | |
| --- | --- | --- | --- | --- | --- | --- | --- | --- | --- | --- | --- | --- | --- | --- | --- | --- |
|  | Day 31 | D32 | D33 | D34 | D-35 | D-36 | D-37 | D-38 | D-39 | D-40 | D-41 | D-42 | D-43 | D-44 | D-45 | D-46 |
| Temperature |  |  |  |  |  |  |  |  |  |  |  |  |  |  |  |  |
| Pulse rate |  |  |  |  |  |  |  |  |  |  |  |  |  |  |  |  |
| BP |  |  |  |  |  |  |  |  |  |  |  |  |  |  |  |  |
| Respiratory Rate |  |  |  |  |  |  |  |  |  |  |  |  |  |  |  |  |
| Oxygen saturation |  |  |  |  |  |  |  |  |  |  |  |  |  |  |  |  |

**Investigations Profile**

| **Trait** | **Value** |  |  |  |  |  |
| --- | --- | --- | --- | --- | --- | --- |
|  | **Admission** | **D-3** | **D-5** | **D-7** | **D-10** | **D-14** |
| **Hb** |  |  |  |  |  |  |
| **WBC** |  |  |  |  |  |  |
| **Neutrophil** |  |  |  |  |  |  |
| **Lymphocyte** |  |  |  |  |  |  |
| **Platelet** |  |  |  |  |  |  |
| **ESR** |  |  |  |  |  |  |
| **CRP** |  |  |  |  |  |  |
| **RBS** |  |  |  |  |  |  |
| **Creatinine** |  |  |  |  |  |  |
| **SGPT** |  |  |  |  |  |  |
| **D-Dimer** |  |  |  |  |  |  |
| **Na** |  |  |  |  |  |  |
| **K** |  |  |  |  |  |  |
| **ECG** |  |  |  |  |  |  |
| **Covid-19**  **RT-PCR** |  |  |  |  |  |  |

**Radio-Imaging**

| **Trait** | **Findings** |
| --- | --- |
| Chest X-Ray  CT Chest | Normal/ Consolidation ( unilateral or bilateral) / Patchy opacity  Consolidation/ Ground Glass Opacity/nodule/ multifocal |

**Post COVID symptoms:**

**Clinical Feature**

| Trait |  | Follow-up  Yes-1, No-2 | | | | | | | | | | | | |
| --- | --- | --- | --- | --- | --- | --- | --- | --- | --- | --- | --- | --- | --- | --- |
|  | On Admission | D | D.. | D.. | D… | D… | D… | D… | D… | D… | D… | D.. | D… | D.. |
| Fever | Yes No |  |  |  |  |  |  |  |  |  |  |  |  |  |
| Cough | Yes No |  |  |  |  |  |  |  |  |  |  |  |  |  |
| Running nose | Yes No |  |  |  |  |  |  |  |  |  |  |  |  |  |
| Sputum | Yes No |  |  |  |  |  |  |  |  |  |  |  |  |  |
| Respiratory distress | Yes No |  |  |  |  |  |  |  |  |  |  |  |  |  |
| Sore throat | Yes No |  |  |  |  |  |  |  |  |  |  |  |  |  |
| Hoarseness of voice | Yes No |  |  |  |  |  |  |  |  |  |  |  |  |  |
| Chest pain | Yes No |  |  |  |  |  |  |  |  |  |  |  |  |  |
| Diarrhoea | Yes No |  |  |  |  |  |  |  |  |  |  |  |  |  |
| Vomiting | Yes No |  |  |  |  |  |  |  |  |  |  |  |  |  |
| Anosmia | Yes No |  |  |  |  |  |  |  |  |  |  |  |  |  |
| Anorexia | Yes No |  |  |  |  |  |  |  |  |  |  |  |  |  |
| Headache | Yes No |  |  |  |  |  |  |  |  |  |  |  |  |  |
| Confusion | Yes No |  |  |  |  |  |  |  |  |  |  |  |  |  |
| others |  |  |  |  |  |  |  |  |  |  |  |  |  |  |
| Others |  |  |  |  |  |  |  |  |  |  |  |  |  |  |
| Others |  |  |  |  |  |  |  |  |  |  |  |  |  |  |

| Trait |  | Follow-up  Yes-1, No-2 | | | | | | | | | | | | |
| --- | --- | --- | --- | --- | --- | --- | --- | --- | --- | --- | --- | --- | --- | --- |
|  | On Admission | D | D.. | D.. | D… | D… | D… | D… | D… | D… | D… | D.. | D… | D.. |
| Fever | Yes No |  |  |  |  |  |  |  |  |  |  |  |  |  |
| Cough | Yes No |  |  |  |  |  |  |  |  |  |  |  |  |  |
| Running nose | Yes No |  |  |  |  |  |  |  |  |  |  |  |  |  |
| Sputum | Yes No |  |  |  |  |  |  |  |  |  |  |  |  |  |
| Respiratory distress | Yes No |  |  |  |  |  |  |  |  |  |  |  |  |  |
| Sore throat | Yes No |  |  |  |  |  |  |  |  |  |  |  |  |  |
| Hoarseness of voice | Yes No |  |  |  |  |  |  |  |  |  |  |  |  |  |
| Chest pain | Yes No |  |  |  |  |  |  |  |  |  |  |  |  |  |
| Diarrhoea | Yes No |  |  |  |  |  |  |  |  |  |  |  |  |  |
| Vomiting | Yes No |  |  |  |  |  |  |  |  |  |  |  |  |  |
| Anosmia | Yes No |  |  |  |  |  |  |  |  |  |  |  |  |  |
| Anorexia | Yes No |  |  |  |  |  |  |  |  |  |  |  |  |  |
| Headache | Yes No |  |  |  |  |  |  |  |  |  |  |  |  |  |
| Confusion | Yes No |  |  |  |  |  |  |  |  |  |  |  |  |  |
| others |  |  |  |  |  |  |  |  |  |  |  |  |  |  |
| Others |  |  |  |  |  |  |  |  |  |  |  |  |  |  |
| Others |  |  |  |  |  |  |  |  |  |  |  |  |  |  |

Principal Investigators: ……………….. Date:
