# Supplementary material for: Post-COVID-19 syndrome among symptomatic COVID-19 patients: A prospective cohort study in a tertiary care center of Bangladesh
Source: PLoS One. 2021 Apr 8;16(4):e0249644. doi: 10.1371/journal.pone.0249644 (PMC8031743; doi:10.1371/journal.pone.0249644)
Supplement: S1 File — (PDF) [file pone.0249644.s002.pdf]

Telephonic interview guide

Post-COVID-19 syndrome among symptomatic COVID-19 patients: A prospective cohort study in a tertiary care center of Bangladesh

Name of the patient

Name of the interviewer

Date of interview

Main focus	Question	Response	Code
Greetings	Assalamualaikum<br><br>How are you?		Response to be written in sentence
Presenting complaints	Do you have fever at present?		Yes -1<br>No-2
	Do you have cough at present?		Yes -1<br>No-2
	Do you have running nose at present?		Yes -1<br>No-2
	Do you have sputum at present?		Yes -1<br>No-2
	Do you have Respiratory distress at present?		Yes -1<br>No-2
	Do you have Sore throat at present?		Yes -1<br>No-2
	Do you have hoarseness of voice at present?		Yes -1<br>No-2
	Do you have chest pain at present?		Yes -1<br>No-2
	Do you have Diarrhoea at present?		Yes -1<br>No-2
	Do you have Vomiting at present?		Yes -1<br>No-2
	Do you have Anosmia at present?		Yes -1<br>No-2

	Do you have Anorexia at present?		
	Do you have Headache at present?		Yes -1<br>No-2
	Do you have any other complain at present?		Response to be written in sentence
Post COVID features Will be asked if patient have clinical recovery (No fever, no cough no respiratory distress)	Have you developed any new complain after recovery?		Yes -1<br>No-2
	What are your new complains?		Response to be written in sentence
	Have you developed exaggeration of previous chronic disease?		Yes -1<br>No-2
	Which of your disease is exaggerated		Response to be written in sentence
	Do you have any minor persisted COVID symptoms?<br>(except fever, cough and respiratory distress)		Yes -1<br>No-2
	Which minor COVID symptoms are persisted?		Response to be written in sentence
Physical findings	What is your Temperature today?		Response to be written in sentence
	What is your oxygen saturation to day		Response to be written in sentence
Concluding remarks	Do you have anything to add?		Response to be written in sentence
	Thank you		

টেলিফোন সাক্ষাতকার নির্দেশিকা

সাক্ষাতকার প্রদানকারীর নাম:

সাক্ষাতকার গ্রহনকারীর নাম:

তারিখ:

গধরহ ভড়পঁং	প্রশ্ন	উত্তর	কোড
সম্ভাষণ	আস্‌সালামুআলাইকুম<br><br>কেমন আছেন?		জবাব লিখিত আকারে লিপিবদ্ধ করুন
উপসর্গসমূহ	আপনার কি এখন জ্বর আছে?		হাঁ -১<br>না-২
	আপনার কি এখন কাশি আছে?		হাঁ -১<br>না-২
	আপনার কি এখন সর্দি আছে?		হাঁ -১<br>না-২
	আপনার কি এখন কফ আছে?		হাঁ -১<br>না-২
	আপনার কি এখন শ্বাসকষ্ট আছে?		হাঁ -১<br>না-২
	আপনার কি এখন গলাব্যাথা আছে?		হাঁ -১<br>না-২
	আপনার কি এখন ফ্যাশ ফ্যাশ কণ্ঠ আছে?		হাঁ -১<br>না-২
	আপনার কি এখন বুকে ব্যাথা আছে?		হাঁ -১<br>না-২
	আপনার কি এখন পাতলা পায়খানা আছে?		হাঁ -১<br>না-২

	আপনার কি এখন বমি আছে?		হাঁ -১<br>না-২
	আপনার কি এখন গন্ধহীনতা আছে?		হাঁ -১<br>না-২
	আপনার কি এখন অরুচিআছে?		হাঁ -১<br>না-২
	আপনার কি এখন মাথা ব্যাথা আছে?		হাঁ -১<br>না-২
	আপনার আর কোন উপসর্গ আছে?		জবাব লিখিত আকারে লিপিবদ্ধ করুন
কোভিড পরবর্তী উপসর্গ (জ্বর, কাশি্ ও শ্বাষকষ্ট না থাকলে)	আপনার কি কোন নতুন উপসর্গ দেখা দিয়েছে?		হাঁ -১<br>না-২
	আপনার কি কি নতুন উপসর্গ দেখা দিয়েছে?		জবাব লিখিত আকারে লিপিবদ্ধ করুন
	আপনার পূর্ববর্তী কোন রোগের উপসর্গ বৃদ্ধি পেয়েছে?		হাঁ -১<br>না-২
	আপনার পূর্ববর্তী কোন রোগের কি কি উপসর্গ বৃদ্ধি পেয়েছে?		জবাব লিখিত আকারে লিপিবদ্ধ করুন
	আপনার কোভিডের সামান্য উপসর্গ এখনো আছে কি?		হাঁ -১<br>না-২
	আপনার কোভিডের সামান্য উপসর্গ কি কি আছে ?		জবাব লিখিত আকারে লিপিবদ্ধ করুন
পরীক্ষণ ফলাফল	আজকের তাপমাত্রা কত ?		জবাব লিখিত আকারে লিপিবদ্ধ করুন
	আজকের অক্সিজেনের মাত্রা কত?		জবাব লিখিত আকারে লিপিবদ্ধ করুন
বিদায়ী মন্তব্য	আপনার কি আর কিছু বলার আছে?		জবাব লিখিত আকারে লিপিবদ্ধ করুন
	ধন্যবাদ		
